# The Onset of Type 2 Diabetes: Proposal for a Multi-Scale Model

**DOI:** 10.2196/resprot.2854

**Published:** 2013-10-31

**Authors:** Filippo Castiglione, Paolo Tieri, Albert De Graaf, Claudio Franceschi, Pietro Liò, Ben Van Ommen, Claudia Mazzà, Alexander Tuchel, Massimo Bernaschi, Clare Samson, Teresa Colombo, Gastone C Castellani, Miriam Capri, Paolo Garagnani, Stefano Salvioli, Viet Anh Nguyen, Ivana Bobeldijk-Pastorova, Shaji Krishnan, Aurelio Cappozzo, Massimo Sacchetti, Micaela Morettini, Marc Ernst

**Affiliations:** ^1^Consiglio Nazionale delle RicercheIstituto per le Applicazioni del Calcolo "Mauro Picone"RomaItaly; ^2^TNO Toegepast Natuurwetenschappelijk OnderzoekMicrobiology and Systems BiologyZeistNetherlands; ^3^Università di BolognaDipartimento di Medicina Specialistica, Diagnostica e SperimentaleBolognaItaly; ^4^University of CambridgeComputer Laboratory, Faculty of Computer Science and TechnologyCambridgeUnited Kingdom; ^5^University of SheffieldDepartment of Mechanical EngineeringSheffieldUnited Kingdom; ^6^University of SheffieldINSIGNEO Institute for in silico MedicineSheffieldUnited Kingdom; ^7^Medisana Space Technologies GMBHDusseldorfGermany; ^8^Università di Roma Foro ItalicoDipartimento di Scienze del Movimento Umano e dello SportRomaItaly

**Keywords:** type 2 diabetes, metaflammation, metabolism, computational biology, simulation, physical activity, multiscale modeling, data integration

## Abstract

**Background:**

Type 2 diabetes mellitus (T2D) is a common age-related disease, and is a major health concern, particularly in developed countries where the population is aging, including Europe. The multi-scale immune system simulator for the onset of type 2 diabetes (MISSION-T2D) is a European Union-funded project that aims to develop and validate an integrated, multilevel, and patient-specific model, incorporating genetic, metabolic, and nutritional data for the simulation and prediction of metabolic and inflammatory processes in the onset and progression of T2D. The project will ultimately provide a tool for diagnosis and clinical decision making that can estimate the risk of developing T2D and predict its progression in response to possible therapies. Recent data showed that T2D and its complications, specifically in the heart, kidney, retina, and feet, should be considered a systemic disease that is sustained by a pervasive, metabolically-driven state of inflammation. Accordingly, there is an urgent need (1) to understand the complex mechanisms underpinning the onset of this disease, and (2) to identify early patient-specific diagnostic parameters and related inflammatory indicators.

**Objective:**

We aim to accomplish this mission by setting up a multi-scale model to study the systemic interactions of the biological mechanisms involved in response to a variety of nutritional and metabolic stimuli and stressors.

**Methods:**

Specifically, we will be studying the biological mechanisms of immunological/inflammatory processes, energy intake/expenditure ratio, and cell cycle rate. The overall architecture of the model will exploit an already established immune system simulator as well as several discrete and continuous mathematical methods for modeling of the processes critically involved in the onset and progression of T2D. We aim to validate the predictions of our models using actual biological and clinical data.

**Results:**

This study was initiated in March 2013 and is expected to be completed by February 2016.

**Conclusions:**

MISSION-T2D aims to pave the way for translating validated multilevel immune-metabolic models into the clinical setting of T2D. This approach will eventually generate predictive biomarkers for this disease from the integration of clinical data with metabolic, nutritional, immune/inflammatory, genetic, and gut microbiota profiles. Eventually, it should prove possible to translate these into cost-effective and mobile-based diagnostic tools.

## Introduction

### Background

Type 2 diabetes mellitus (T2D) is a metabolic disorder of late adulthood and old age, characterized by a decrease in glucose uptake from the blood, resulting in hyperglycemia (high blood sugar levels). In some individuals, this leads to a physiological condition referred to as “insulin resistance” in which the hormone insulin becomes less effective at lowering blood sugar. Insulin resistance can lead to insufficient production of insulin by the pancreatic β-cells and to the clinical condition known as T2D. Both insulin resistance and T2D are associated with obesity, aging, and inactivity [1].

The prevalence of diabetes, and particularly type 2, has increased markedly over the last 50 years in parallel with increasing rates of physical inactivity and obesity. Type 2 diabetes now accounts for about 90% of total cases of diabetes worldwide. As of 2010, there are approximately 285 million people in the world who are diagnosed with T2D compared to around 30 million in 1985 [1]. The figures within the European Union (EU) are equally stark: the World Health Organization states that more than 50 million European citizens are currently affected [2]. The epidemic growth of obesity in Europe, its aging population, and the often sedentary lifestyle of its citizens have led to an explosion in the incidence of T2D. Based on the assumption in this report that approximately 50% of affected people are unaware of their disease, it can be estimated that approximately 60 million people in Europe are likely to be affected by T2D.

An emerging view attributes the main driving forces of diabetes development to chronic energy overload from excess of nutrition, metabolic imbalance, reduction of metabolic flexibility, and inflammation. The role of a pervasive and multi-systemic state of inflammation triggered by the immune system, is also endorsed by 17 clinical trials of therapies that use anti-inflammatory approaches to treat T2D or prediabetic states. Several of these have already reached conclusive results, confirming the role of inflammation in the pathogenesis of T2D [1].

The contributions of mechanisms at the molecular, tissue, and organ levels to the physiological processes that lead from obesity to insulin resistance to the full-blown disease are intertwined in a complex and strongly patient-specific manner, suggesting that T2D must be considered from a personalized and systemic medicine perspective. The multi-scale immune system simulator for the onset of type 2 diabetes (MISSION-T2D) aims to develop personalized diagnostic markers based on the systems view.

In this complex scenario, there is an urgent need for efficient predictive approaches and models, capable of identifying a set of biological characteristics suitable for large-scale, cost-effective screening campaigns that aim to (1) drastically reduce the number of undiagnosed cases; (2) detect T2D at very early stages, at which point the disease appears to be still reversible; and (3) stratify cases in order to predict and, if possible, counteract T2D complications before they start exerting their detrimental effects. Thus, the identification of such a set of metabolic and inflammatory characteristics (ie, model-generated biomarkers and algorithms) and the implementation of their detection on user-friendly, cost-effective devices that can be used by primary care physicians or the patients themselves are the major goals of MISSION-T2D.

From a technical point of view, this study will implement a process workflow to simulate the metabolic and immune responses of tissues, particularly adipose tissues and pancreatic islets, to nutrition intake and metabolic alterations. This model will take into account patient-specificity through parameters derived from metabolic flexibility profiles, lifestyle parameters, nutritional habits and genetic signatures. The integrated modeling platform resulting from the above process will be the basis for a user-friendly diagnostic tool, ultimately developing a mobile app for Android, iOS, or Windows Mobile to be used by physicians for patient-specific intervention, which we will call the “physician assistant”, and will be known as “personal coach” by patients to monitor their metabolic health status.

This project is supported by the Virtual Physiological Human (VPH) initiative, which is an EU Framework Seven funded program that aims to develop a framework of computational biomedicine methods and technologies to investigate the human body as a whole [3].

### Type 2 Diabetes as a Systemic Inflammatory Disease

**Figure 1:**
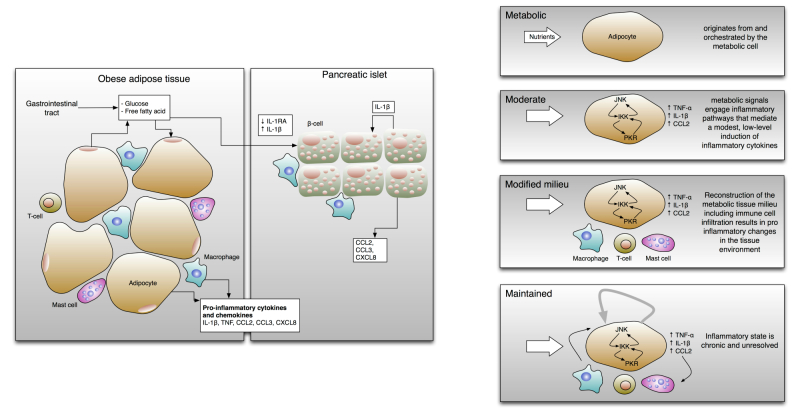
Left: Excessive levels of nutrients, including glucose and free fatty acids, stress the pancreatic islets and insulin-sensitive tissues such as adipose tissue, leading to the local production and release of cytokines and chemokines (eg, IL-1β, TNF, CCL2, CCL3, and CXCL8). Furthermore, production of IL-1 receptor antagonist (IL-1RA) by β-cells is decreased. As a result, immune cells will be recruited, which contribute to tissue inflammation [14]. Right: Hallmarks of metaflammation. The first feature of this type of inflammation in obese individuals is that it originates from signals within metabolic cells such as adipocytes. Second, the metabolic signals trigger inflammatory intracellular signaling pathways that mediate downstream inflammatory responses (eg, JNK, IKK, or PKR pathways). The activation of these mediators induces a low level of chronic inflammation in response to the excess nutrients. Over time, this may induce the recruitment and activation of specialized immune cells [1].

Different interlinked mechanisms participate in the onset of T2D, with pancreatic dysfunction as the major cause. In response to the development of insulin resistance, the specialized cells that are devoted to insulin production (ie, the β-cells in the pancreatic islets) react by increasing their cell mass and amount of insulin secretion (see Figure 1). However, when the functional expansion of islet β-cells fails to compensate for the degree of insulin resistance, insulin deficiency and ultimately T2D may develop. Thus, the onset and progression of T2D are determined by the progressive failure of β-cells to produce sufficient levels of insulin [4,5].

Many insulin-resistant individuals do not become diabetic since their β-cells are able to compensate for the increased demand for insulin. Only about one-third of all people with insulin resistance will develop chronic hyperglycemia and eventually T2D. Progression to disease development involves excessive calorie intake, chronic energy overload, imbalance of metabolic pathways, impairment of metabolic flexibility, and related immune dysfunction. Metabolic flexibility is defined as the capacity to utilize diverse metabolic pathways for the uptake and storage of tissue energy from lipids and carbohydrates. Metabolic flexibility is lost in obese and diabetic individuals [6,7]. Obesity is thought to be among the primary causes of T2D, although genetic and epigenetic factors are likely to play an important role.

It has been suggested that a number of possible stress mechanisms (referred to here as “stressors”) lead to insulin resistance and β-cell dysfunction, and play a part with them in the development of the full complex T2D phenotype. These include oxidative stress, endoplasmic reticulum stress, deposition of amyloid (ie, insoluble fibrous proteins) in the pancreas, and deposition of ectopic lipid in the muscle, liver, and pancreas [8,9].

All of these stressors can be caused by overnutrition, although it has been difficult to determine which mechanisms are the most important in each tissue, or how much they differ between individuals with T2D. However, it is noteworthy that each of these cellular stressors is thought either to induce an inflammatory response by itself or to be exacerbated by or associated with inflammation [10,11]. Inflammation is a complex, systemic, multi-scale physiological process that is necessary to cope with damaging agents and is fundamental for survival, involving a variety of cells, organs, and systems. The complexity of the inflammatory process escapes reductionist and linear approaches, since it is characterized by nonproportional kinetics as well as by numerous and nested feedback loops [12]. Ultimately, T2D can be conceptualized as being derived from a chronic inflammatory state that is initiated by an excess of nutrients and referred to as metabolic inflammation or metaflammation (see Figure 1). Proof-of-concept clinical studies have demonstrated the potential use of an anti-inflammatory molecule in T2D therapy [13].

The long-term consequences of T2D for most patients are severe. These include both macrovascular complications, including atherosclerosis, cardiovascular diseases, and amputations; and microvascular complications, including retinopathy, nephropathy, and neuropathy.

A number of possible stress mechanisms that are linked to inflammation and have been hypothesized to explain insulin resistance and β-cell dysfunction have already been mentioned. Metaflammation (ie, metabolic inflammation) is the most recent conceptualization in the field of metabolic diseases such as obesity and T2D [14], suggests that the hallmark of these pathologies is a chronic, sterile inflammatory state that is initiated by an excess of nutrients. This, and excessive levels of glucose and free fatty acids (FFAs), stresses the pancreatic islets, and therefore the β-cells and insulin-sensitive tissues such as adipose tissue, liver, and muscle, leading to the local production and release of immune inflammatory mediators such as cytokines and chemokines [1].

The immune system plays a pivotal role in the outbreak of the inflammatory state. Activated immune cells such as macrophages and T-cells invade adipose tissue, which is the site where these inflammatory alterations were first described and have been most studied. Consequently, the adipose tissue alters in response to this inflammation, triggering specific cellular programs that activate multiple signaling networks resulting in the production of a variety of inflammatory compounds. Furthermore, the activation of inflammatory pathways such as nuclear factor κB (NF-κB), protein kinase RNA-activated (PKR) and c-Jun N-terminal kinases (JNKs) pathways also occurs in liver cells, and muscles are deeply influenced by peripheral inflammation.

To summarize, metaflammation is metabolic, moderate, and characterized by local expression of inflammatory mediators. These are induced by stress-sensing cellular mechanisms originated through signaling pathways involving the inhibitor of κB kinases (IKKs) and JNK kinases, and a range of other signaling molecules that have been collectively termed the “inflammasome”. This peculiar type of inflammation, unlike the classic inflammatory paradigm, is associated with a reduced metabolic rate**,** activates specialized immune cells, and creates a modified milieu in which an altered composition of immune cells favors a proinflammatory tissue environment that is maintained without apparent resolution (see Figure 1).

There is a second inflammatory component that must be considered: the interaction between the inflammatory process and aging. The aging process itself is accompanied by a chronic low-grade inflammation, which has been termed “inflammaging”, a term derived from inflammation and aging. As it is known that insulin resistance and T2D are associated with aging, it is likely that the combination of metabolic-driven and age-driven inflammatory pathways plays a pivotal role in T2D pathogenesis.

**Figure 2:**
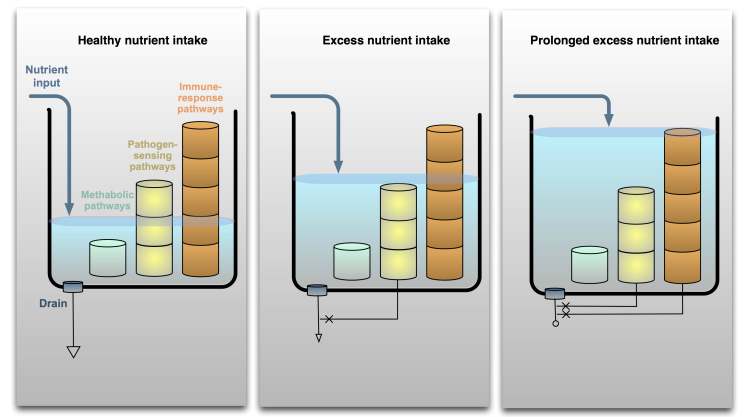
Nutrient overload signals spill from metabolic pathways to immune-response pathways. Nutrient input under normal or healthy conditions should engage metabolic pathways within the cells, leaving immune-response pathways inactive. With increased nutrient intake, the levels of nutrients flooding the system may rise enough that the overflow stimulates pathogen-sensing pathways. Because these pathways recognize biological molecules such as specific fatty acids, excessive amounts of nutrient moieties may also be able to activate such sensors. Once the immune sensors are activated, they may be antagonistic to the metabolic pathways, in effect blocking the drain of nutrient metabolism. If the nutrient excess persists to an extreme state, immune-response pathways of specialized immune cells may also be activated. The involvement of these pathways will intensify the inhibition of metabolic pathways and contribute to the backlog of nutrients in the system [14].

Metaflammation has inhibitory effects on insulin action through inflammatory kinases (eg, JNK, IKK, and PKR) in metabolic tissues (eg, adipocytes) and disrupts nutrients and energy metabolism (see Figure 1, right). Metaflammation is also characterized by the activation of the classical pathogen-sensing or immune response pathways by stimulation of the toll-like receptors (TLR) as a result of an excess of nutrients [12] (see Figure 2). This observation suggests that aging-related and metabolic inflammation (inflammaging and metaflammation) can share stimuli and pathogenic mechanisms.

In this context, it is important to note that inflammatory stimuli derived from intestinal bacterial flora (gut microbiota) can make a substantial contribution to inflammaging as well as to metaflammation and obesity. Several studies on animals and humans have shown that populations of gut microbiota differ significantly between average-weight and obese individuals [15]. Moreover, a recent study has shown that the composition of the gut microbitota alters with age, and that there is a correlation between age-related changes in micobiota and increased levels of inflammatory cytokines such as interleukin 6 (IL-6) and interleukin 8 (IL-8) in the plasma [16,17].

Another factor that plays a non-trivial role in maintaining metabolic balance is physical activity. Epidemiological studies suggest that physically active individuals have a 30-50% lower risk of developing type 2 diabetes. Physical activity, measured in terms of exercise intensity (eg, the number of steps walked, kilocalories burned per minute, or metabolic equivalent units) impacts the regulation of body weight, body mass index, insulin resistance and sensitivity, glycemic control, atherogenic dyslipidemia, and inﬂammation [18].

Taken as a whole, most of the mechanisms that underpin these complex interactions and circuits are still poorly understood. An integrated modeling approach such as that proposed in this project is thus necessary to grasp, as well as to model, the complexity of the pathological conditions that lead to T2D.

## Methods

###  A Multi-Scale Model for T2D (Concepts and Architecture of MISSION-T2D)

In this study, we intend to take a practical approach towards addressing the typical situation in which a person presents her/himself to the general practitioner with one or more signs of the onset of insulin resistance or diabetes, such as high waist circumference, elevated sugar blood levels (obtained through the fasting plasma glucose test), and/or elevated plasma triglycerides. The doctor will then need to ask what the best treatment schema for the particular person is (ie, which treatment will lead to the best results, with the fewest side effects, and in the shortest time). This might be a proposed lifestyle intervention, a drug treatment, or both. The optimum solution differs from patient to patient; therefore, in order to solve this problem the doctor will need to look at the patient’s individual condition and perform a diagnosis from a systems perspective. Adopting the systems view acknowledges that any local change in a physiological system affected by an intervention will have repercussions on other parts of the system. For example, if a certain intervention merely blocks lipid accumulation in the liver, the lipid will start to accumulate and perhaps cause problems elsewhere, such as the heart muscle.

The picture is further complicated by patient-specific genetic, metabolic, and lifestyle traits that promote the stress-induced production of inflammatory mediators such as IL-1ß, TNF, chemokine (C-C motif) ligand 2 (CCL2), CCL3, and (C-X-C motif) ligand 8 (CXCL8). These inflammatory mediators will trigger the innate immune system, finally leading to a chronic low-grade inflammatory state (ie, tissue inflammation). This inflammation will lead to reduced insulin signaling (ie, insulin resistance in tissues and organs). The resulting need for increased insulin production will put pancreatic ß-cells under increasing chronic stress, which will induce a specific inflammation state impairing insulin production from pancreatic ß-cell islets. Finally, this will lead to more severe metabolic disturbances and to chronic hyperglycemia as a precursor of T2D.

In each case, the decision about which interventions to perform must take into account the effects that will arise due to interactions between different organs and processes in the body. To enable this, the diagnosis must take into account the actual level of flexibility of these same processes and organs, (ie, their capacity to cope with metabolic and inflammatory challenges). Thus, on the one hand, a diagnostic tool must be able to predict which processes are in fact at risk of reaching the limits of their capacity. On the other hand, it must predict which intervention(s) will be necessary to shift the metabolic/inflammatory demands in that individual to processes and organs that still have sufficient capacity, while at the same time reducing the overall metabolic/inflammatory load on the system.

**Figure 3:**
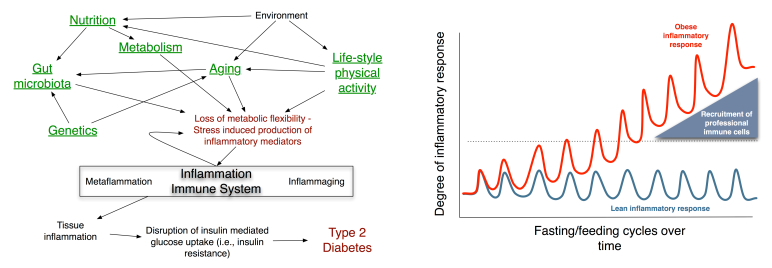
Left: Relationships between the concepts to be modeled in MISSION-T2D: Each aspect will be taken into account either through empirical data or via modeling, with submodels built up into a single integrated model. This will take as input information from genetics, nutrition, age, physical activity habits, and gut microbiota to elaborate a patient-specific risk assessment for the onset of T2D. Right: The pulsatile inflammatory response during feeding, showing differences between the responses of normal and obese individuals’ reactions over time: Fasting/feeding cycles induce low-level inflammatory responses in the metabolic cells of average-weight, healthy individuals that are easily resolved. During the high-fat diet or excess feeding that results in obesity, responses to food become more intense and frequent, and the resolution of the inflammatory response becomes less efficient, raising the baseline level of inflammation in metabolic tissues. Once the level of inflammatory response reaches a certain threshold in the metabolic cells, professional immune cells are recruited and activated. The participation of these cells in the inflammatory response alters the tissue environment toward a proinflammatory milieu and exacerbates the inflammation even further [14].

The left panel of Figure 3 describes the relationships between different concepts that we will be considering in MISSION-T2D. The model inputs that take patient specificity into account are underlined in the diagram. These inputs include genetic traits (eg, variants of genes such as the immune cell receptor-coding gene CD44 and SPP1 (secreted phosphoprotein 1) [19], nutrition habits (eg, markers of glucose and free fatty acid intake), lifestyle habits (eg, physical activity), age, and genetic sampling of the gut microflora (measured by a human intestinal trait chip or HITchip).

At present, many types of data that are known or of potential relevance to this project are available in clinical or research settings. These include genomics, transcriptomics, metabolomics, proteomics, epigenetics data, and miRNAs, as well as variables derived from more classical physiology and clinical chemistry, and also from medical imaging. Relevant parameters that can be determined by imaging include the thickness of the inner layers of the arterial wall. The important question to answer is how these data can best be used to model different biological processes that relate to an individual’s risk of developing type 2 diabetes, and then to select among the relevant variables the most suitable for implementation in cost-effective diagnostic or personalized digital coaching tools.

We understand that a wide variety of measured variables are involved in many different biological processes that are integrated on different scales of time and space (ie, from molecular to cells, tissues, organs, and the whole body). A practical solution to deal with the high number of factors involved and the complexity of interactions between the inflammatory and metabolic processes in several organs, and across many orders of magnitude of space and time scales, involves the integration of interconnected models at different aggregation levels of biological processes, roughly corresponding to these different spatial and temporal scales [20].

In this study we will therefore apply a novel multi-scale computational approach to model inflammation in type 2 diabetes. We will develop a composite simulation system embracing four levels of mathematical descriptions covering: (1) the intra-cellular metabolic and gene expression level, (2) the cellular level involving the dynamics of the immune system cells, (3) the organ level, and (4) the disease process within the whole body. Different mathematical and computational models will map aspects of physiology and pathology that are represented at each of these levels. The outputs of each model will directly feed the variables and/or the parameters of the next aggregated model so that it will be eventually possible to merge them all into a single workflow or multi-scale integrated simulation system (see Figure 5).

The models to be developed using MISSION-T2D will involve the following scales: First, the microscopic intracellular scale, or the molecular level, which links to cellular genome-scale networks (ie, the highest level of process detail). This type of modeling typically involves top-down statistical analyses of -omics responses to external stimuli which can be used to determine the subset of pathways and/or network modules that specifically respond to factors of interest and preselect them for bottom-up mechanistic modeling. Molecular scale models to be developed within MISSION-T2D will describe the cellular metabolism of ß-cells and macrophages and the transduction of nutrient components to stimulate the immune system and lead to the inflammatory state, focusing on gut-related processes and the onset of stress-induced inflammation. Second, the mesoscopic intercellular scale, or the tissue/plasma level, which links to the dynamics of tissue/organ-aggregated metabolic and inflammatory processes and plasma markers. These markers change on time scales that range roughly from minutes to days, and the data to calibrate models at this level are typically taken from challenge tests. Models to be developed at this level will involve the dynamic effects of nutrients on the immune system, and specifically the elicitation of a nonspecific inflammatory state leading to a full-blown activation of the adaptive (ie, specific) immune system response. Third, the macroscopic scale, or the organ level, which refers to the main organs affected by T2D, such as the gut and the adipose tissue. Processes to be modeled at this level will involve the triggering of the immune system, and specifically how different types of immune cells (eg, lymphocytes) and organs (eg, lymph nodes) communicate to start the immune-specific response. Data to calibrate models at this level are typically taken from diet/lifestyle/drug intervention studies. In this case the inter-organ process balance level will link the descriptors of integrated and aggregated effects of the nutrient-stimulated inflammation processes that have been defined at the mesoscopic scale roughly on a time scale of days to months. Finally, the whole body disease process level, which links to disease risk. Models developed on this scale will integrate and aggregate the disease-mediating effects of the variables measured or modeled at the macroscopic scale, typically on the multi-year time scale. Data to calibrate these models are typically taken from cohort studies. We will analyze the effect of model-predicted lifestyle interventions on parameters at the macroscopic scale, including physical activity, and will combine them with established clinical methods of T2D risk assessment. Therefore, we will be able to validate the predictions of our integrated model and propose new predictive reporter variables that can be applied to give feedback on T2D risk status, in both clinical and personal settings.

**Figure 4:**
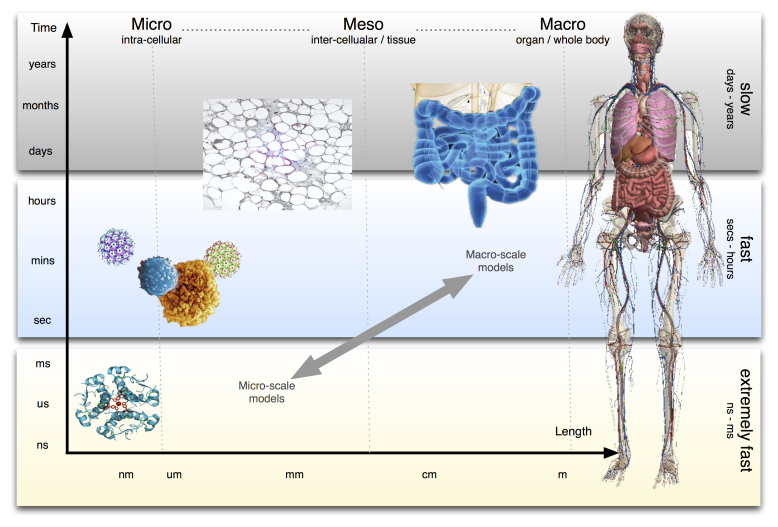
T2D is a complex disease and its onset and progression cover different space-time scales, from molecular events (milliseconds) up to organ deterioration (years), taking into account the contribution of the individual genetic traits. MISSION-T2D project aims to cover and integrate models at all these levels, fully supporting the VPH initiative that aims to develop a computational framework to investigate the human body as a whole in health and disease [2].

The novelty of our approach lies in the integration of various sources of biological data, and processing them by using mathematical and simulation tools, similar to the way nutrition and lifestyle habits are “processed” by the human body. The result of this processing is a physiological condition that can in certain cases become pathological. The approach is systemic, as we will include various levels of description. The final outcome of the integrated modeling platform will cover different space-time scales involved in the complex process of the T2D onset and progression (Figure 4), from the molecular event (milliseconds) up to organ deterioration (years), taking into account the contribution of genetics to nutritional habits.

This gene regulatory network model will be taken from literature [21] (see Figure 6 for comparison) and simulated as a Boolean (gene regulatory) network using the described techniques [22]. The macroscopic scale will then be represented by macroscopic observables such as the number of infiltrated macrophages in adipose tissue or the overall levels of insulin.

**Figure 5:**
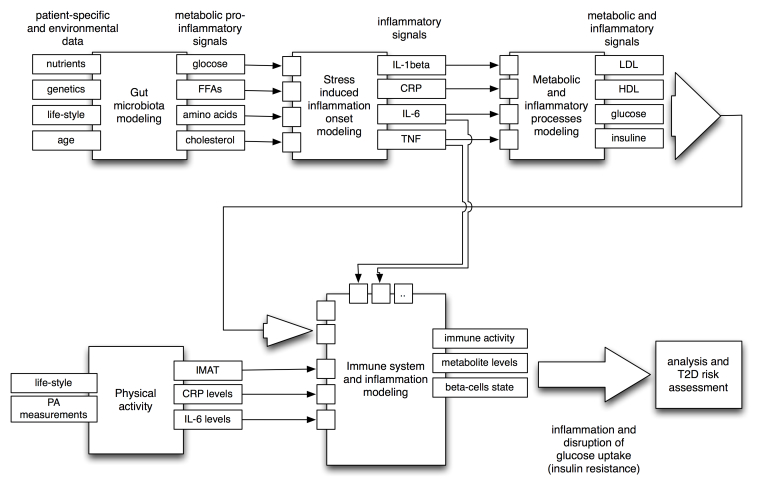
A simplified systemic view of the models to be developed within MISSION-T2D and the interdependencies between them. Input-output relationships among modules of these models are depicted. Feedback (eg, IL-6 from the immune system to the stress-induced model) is not shown for simplicity.

**Figure 6:**
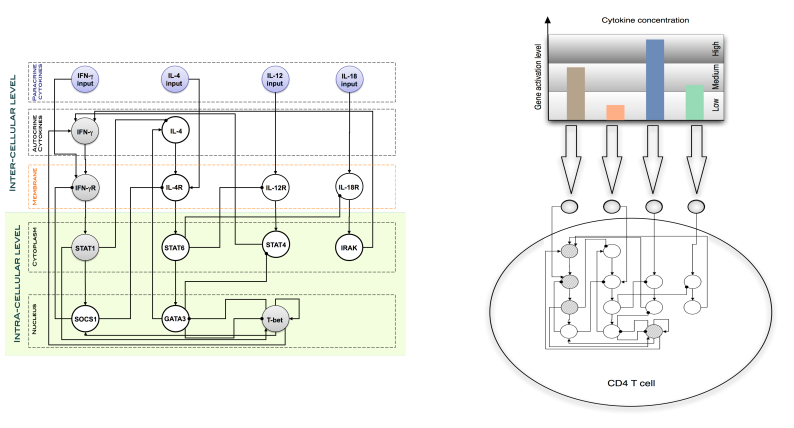
Left: Each helper T lymphocyte (Th cell) is equipped with an intra-cellular dynamics consisting of a gene regulatory network to describe the dynamical rule for the differentiation from Th0 to Th1/Th2 phenotype. Right: The regulatory network dynamics is a function of the cytokine environment [22].

### The Integrated Scientific and Technological Platform of MISSION-T2D

The aim of MISSION-T2D is to be able to validate in silico the potential approach of targeting inflammatory mediators as a treatment for T2D, and to support a causative role for inflammation in the pathogenesis of the illness using quantitative data.

The integrated modeling platform to be developed using MISSION-T2D will offer an opportunity to qualitatively and quantitatively model the effect of different treatments and clinical approaches on various systemic features of the disease simultaneously. These will include defective insulin secretion by β-cells, insulin resistance in adipose tissue, metabolic parameters, and vascular complications among others. We will model the effects of using anti-inflammatory drugs, either alone or in combination, and will modify individuals’ nutritional habits and other behavior such as physical activity, and will evaluate in silico the capability of each single factor to alter the course of the disease.

MISSION-T2D is an integrated model which will allow users to assess the efficacy of anti-inflammatory approaches for improving glycaemia and lessening the complications of T2D, and the durability of their effect. The integrated model platform that we plan to develop will also be able to give insight into the best therapeutic modalities, including whether life-long treatment or short-term interventions are better at breaking inflammatory flares. The model will provide quantitative data about anti-inflammatory strategies that target the underlying mechanisms of the disease, aiming to provide clinical guidance on how and when to start specific therapies in order to prevent the progression of early disease, or to impinge on the overt manifestation of the disease. These models will also provide indications for various immunomodulation strategies in T2D, including whether they will be well-tolerated, which side effects will occur and which drawbacks of immunosuppression will be present.

**Figure 7:**
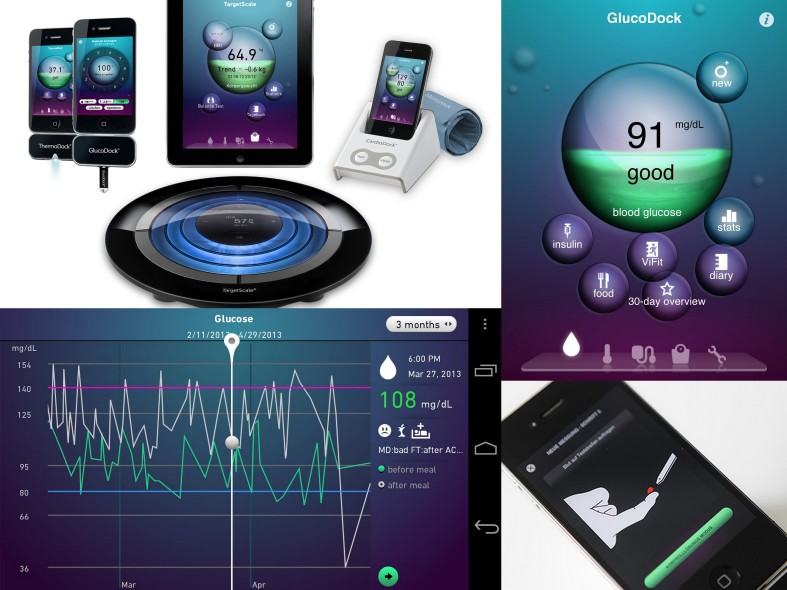
Sensor technology and the use of portable communication devices offer the possibility of storing and accessing data from our daily life to improve self-knowledge. Insights gained by performing these measurements can be used, for example, to change life-threatening habits, adopt a healthier lifestyle, or take more informed treatment decisions.

This approach, once validated, will provide a valuable tool for the physician in the diagnostics and treatment of this disease. Furthermore, our models will provide emotionally engaging, real-time, personalized, nutritional, and behavioral guidance as well as health tracking for individual patients (see Figure 7).

The definition of model structures at different levels will ensure that the models that are to be created or adapted from other published scenarios will be interpretable; and therefore, quantifiable at each individual level since the number of model variables at each level will remain limited. The coherence between models at different time scales (ie, strictly linking outputs at a given level with variables at the next higher level) will lead to a true systems model that is able to effectively span the different space and time scales involved. The overall architecture will translate input information (eg, derived from challenge tests, activity measurements, and baseline plasma analyses) onto the physical condition of a patient, lifestyle, and nutrition habits, and provide information that can be used for practical diagnostic and/or predictive purposes at a personalized level.

Therefore, the systems approach will effectively enable us to build disease risk prediction models by linking diabetes development (at the whole body level) to metaflammation-induced variation in insulin resistance on a time scale of weeks or months (at the macroscopic level), to dynamic processes in inflammation and metabolism at a time scale of hours or days (at the mesoscopic level), and to molecular details (at the microscopic level).

This integrated model will allow the simulation of lifestyle and nutrition habits of individual patients with specific genetic and metabolic characteristics for prolonged periods of time, thus effectively forecasting their risk of developing full-blown T2D. In other words, it will take as its input the cycles of fasting and feeding that induce the low-level inflammatory responses in metabolic cells that are easily resolved in average-weight and healthy individuals; however, it induces a less efficient resolution of the inflammatory response. in obese individuals with excessive or high-fat diets. This raises the baseline of inflammation in metabolic tissues and once the level of inflammatory response in the metabolic cells reaches a certain threshold, professional immune cells are recruited and activated, exacerbating the inflammation even further potentially leading to overt disease [14].

## Results

This study was initiated in March 2013 and is expected to be completed by February 2016.

## Discussion

The aim of MISSION-T2D is to construct a predictive computer-based simulation tool for the development of type 2 diabetes. This tool will have three characteristics. First, it will be patient-specific. We will use clinical data (eg, blood sample parameters, age), genetic traits (eg, variants of immune specific genes such as CD44 and SPP1), nutrition habits (eg, glucose and free fatty acid intake), lifestyle habits (eg, physical activity) and a gut microbiota genetic sampling (measured through eg, HITchip) to build a patient-specific profile to be used as to set up the parameters and initial conditions of the integrated simulator. Second, it will be predictive. The integrated workflow will elaborate on the patient-specific parameters and perform a risk assessment for the onset of T2D in the individual concerned. Third, it will integrate medical, biological, and environmental data. The models will integrate environmental factors (nutrition and lifestyle habits) with medical (clinical tests results) and biological factors (pertinent genetic traits and gut microbiota), enabling the development of a predictive model for understanding the pathogenesis and the progression of type 2 diabetes. Moreover, the simulations will allow users to investigate the influence of other health factors such as age and physical exercise with the onset and evolution of the disease.

## Abbreviations

CCL2: chemokine (C-C motif) ligand 2
CXCL8: chemokine (C-X-C motif) ligand 8
EU: European Union
FFA: free fatty acid
HITchip: human intestinal trait chip
IKKs: inhibitor of kB kinases
IL: interleukin
JNKs: c-Jun N-terminal kinases
MISSION-T2D: The multi-scale immune system simulator for the onset of type 2 diabetes
NF- κB: nuclear factor kappa-light-chain-enhancer of activated B cells
PKR: protein kinase RNA-activated
T2D: Type 2 diabetes mellitus
TLR: toll-like receptors
VPH: Virtual Physiological Human
